# Supplementary figures and images for: Routine screening for SARS CoV-2 in unselected pregnant women at delivery
Source: PLoS One. 2020 Sep 29;15(9):e0239887. doi: 10.1371/journal.pone.0239887 (PMC7524006; doi:10.1371/journal.pone.0239887)

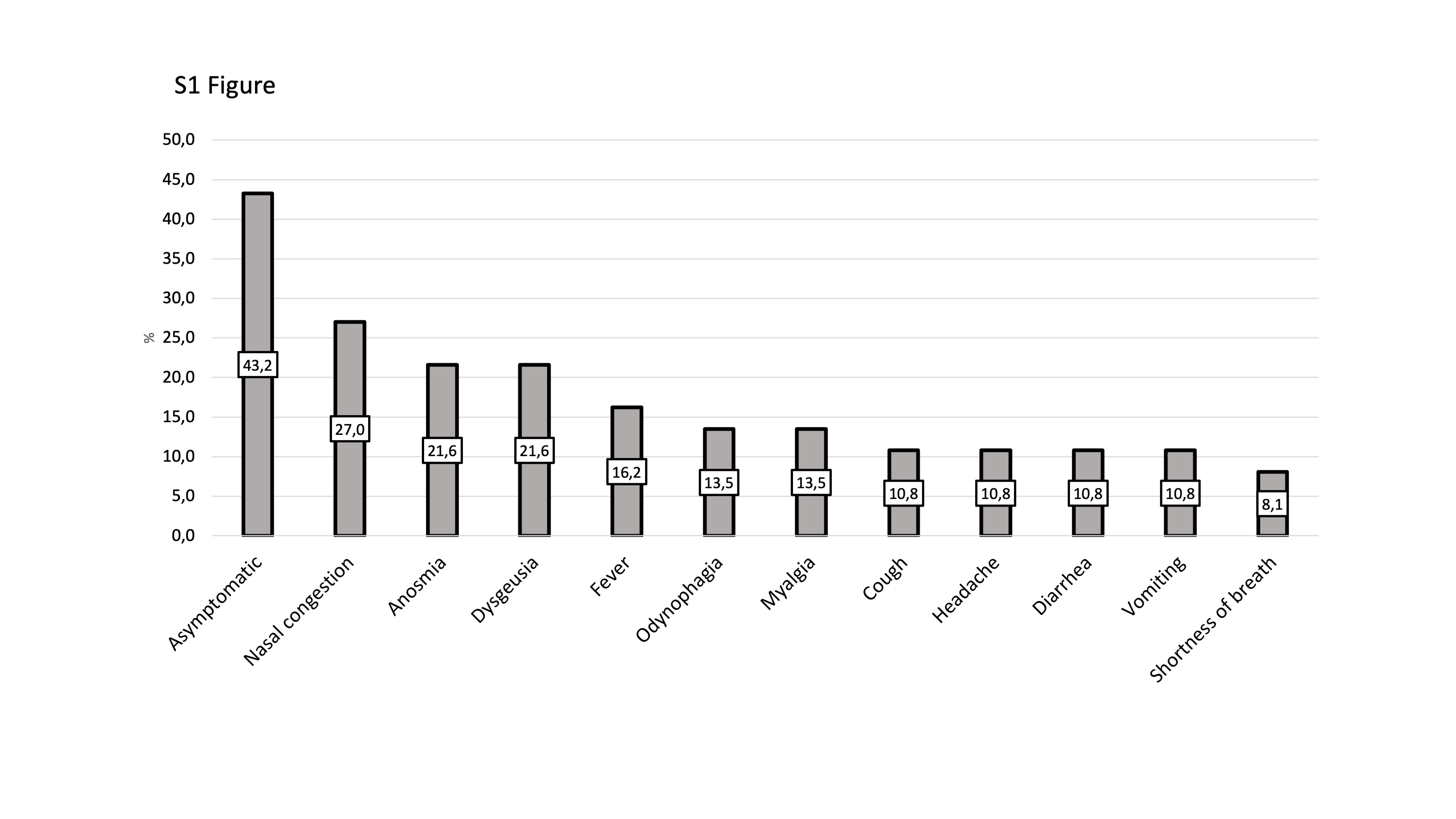

Supplement: S1 Fig — (TIFF) [file pone.0239887.s001.tiff]
