# Supplementary material for: Routine screening for SARS CoV-2 in unselected pregnant women at delivery
Source: PLoS One. 2020 Sep 29;15(9):e0239887. doi: 10.1371/journal.pone.0239887 (PMC7524006; doi:10.1371/journal.pone.0239887)
Supplement: S1 Appendix — (DOCX) [file pone.0239887.s002.docx]

**S1 Appendix:** Entry survey (For confirmed cases)

| ID: |
| --- |
| Diagnostic test sample collection date: dd/mm/yyyy |
| Symptoms:  o Fever ≥ 37.8  o Cough  o Headache  o Shortness of breath  o Myalgia  o Odynophagia  o Nasal congestion  o Digestive symptoms (diarrhea / vomiting)  o Anosmia  o Dysgeusia  o Anorexy  o None  Other: ____ |
| Delivery data:  · Gestational age (weeks + days):  · Weight (grams):  · Apgar 1 min:  · Apgar 5 min: |
| Newborn data:  · Neonatology admission:  o Yes  o No  · Positive PCR for SARS-CoV-2:  o Yes  o No  o Pending test result |

**S1 Appendix (Spanish version): Encuestra de ingreso (Casos confirmados)**

| RUT: |
| --- |
| Fecha de toma de test diagnósticos: dd/mm/aaaa |
| Síntomas  o Fiebre ≥ 37.8  o Tos  o Cefalea  o Disnea  o Mialgia  o Odinofagia  o Congestión nasal  o Síntomas digestivos (diarrea / vómitos)  o Anosmia  o Disgeusia  o Anorexia  o Ninguna  Otro: ____ |
| Información del parto:  · Edad gestacional (semanas + días)  · Peso (gramos)  · Apgar 1 min:  · Apgar 5 min: |
| Información del recién nacido:  · Ingreso a unidad de neonatología:  o Si  o No  · PCR positiva para SARS-CoV-2:  o Si  o No  o Resultado pendiente del test |
