# Supplementary material for: Routine screening for SARS CoV-2 in unselected pregnant women at delivery
Source: PLoS One. 2020 Sep 29;15(9):e0239887. doi: 10.1371/journal.pone.0239887 (PMC7524006; doi:10.1371/journal.pone.0239887)
Supplement: S1 Table — (DOCX) [file pone.0239887.s003.docx]

S1 Table: maternal and pregnancy outcomes, according to presence of clinical symptoms in patients with positive RT-PCR for SARS CoV-2 Infection.

|  | Asymptomatic Cases.  (N=16) | Symptomatic Cases.  (N=21) | P-value. |
| --- | --- | --- | --- |
| Gestational age at delivery (weeks) | 39.1 (± 0.9) | 38.1 (± 2.3) | 0.133 |
| Preterm birth | 0 (0.0) | 4 (19.1) | - |
| Birthweight (grams) | 3433 (± 446) | 3078 (± 551) | 0.125 |
| Small for gestational age | 0 (0.0) | 2 (9.5) | - |
| Caesarean delivery | 8 (50.0) | 10 (47.6) | 0.886 |
| Instrumental vaginal delivery | 1 (6.3) | 0 (0.0) | - |
| 5th minute Apgar Score ≤ 7 | 0 (0.0) | 1 (5.0) | - |
| NICU Hospitalisation | 3 (18.8) | 2 (9.5) | 0.416 |
| Perinatal death | 1 (6.25) | 0 (0.0) | - |
| Data is presented as: means (± standard deviations) or absolute frequencies (%) | | | |
