# Supplementary material for: Routine screening for SARS CoV-2 in unselected pregnant women at delivery
Source: PLoS One. 2020 Sep 29;15(9):e0239887. doi: 10.1371/journal.pone.0239887 (PMC7524006; doi:10.1371/journal.pone.0239887)
Supplement: Attachment — Submitted filename: Response to reviewers 2.docx [file pone.0239887.s005.docx]

**Response to Editor / Reviewers comments**

Dear Editor, please find below and itemized answers to all the reviewers queries according to journal requested format.

**Reviewer #3:** *Most of this referee's requests have been considered. However, the discussion still appears too weak. If the final message of the authors is to propose a universal screening for SARS-CoV-2 in pregnancy in endemic area, it is essential to compare in depth with other epidemiological realities as already suggested. Moreover manuscripts reported in my previous review with important epidemiological data have not been considered in this revised paper (DOI: 10.1016/j.ajogmf.2020.100118, DOI: 10.1002/jmv.26267, DOI:10.1002/ijgo.13162), but again, if the aim is to propose a universal screening for COVID-19 in pregnancy, I believe it is essential to mention and discuss them in order to provide readers of this paper a better idea of the global picture on the pandemic in pregnancy.*

Reviewer suggestion has been considered and changes have been made according to recommendations across the manuscript in order to strengthen our arguments and emphasize the role of universal screening among obstetric population. Regarding comparison to previous reports it’s our believe that a full “in dept” analysis of pandemic situation between different geographic areas is beyond the scope of our study as several complex factors should be taken into account to proper comparison, such as social dynamics, population healthcare access or government policies. It’s true that some of the recommended references by the reviewer (Cosma S, et al.) compare different realities thought accumulative incidence, however the former is this directly affected by testing number. Nonetheless, in order to allow the reader a better interpretation of reported results the following paragraph has been added into the discussion section:

“It could be argued that previous reports on universal screening in obstetrics population do not provide information on the local situation of the pandemic, so it is difficult to estimate the real implications and external validity of their findings. Moreover, as it has been stated, the different time points of patient recruitment along with the rising and falling phase of the pandemic curve, explain the different rates of positive RT‐PCR results.[22] Therefore, in areas with high prevalence of infection, it could be expected that more women may be positive but asymptomatic.[23] This could be seen in reports by Sutton et al.[10], Vintzileos et al.[9], Bianco et al.[13] and Dória et al.[16] All of them were conducted in areas and timepoints with reported high prevalence of infection,[24] and showed higher observed SARS-CoV-2 infection prevalence, ranging between 11 and 19%, with up to 15% of asymptomatic confirmed cases among screened population. On the other side, reports by Naqvi et al.[25] and Gagliardi et al.[12], reported a low performance of universal screening based either on an overall lower disease burden in their region or due to a referred “steady state” of virus circulation, with less than 1% of asymptomatic confirmed cases. In an intermediate epidemiological situation reports by Khalil et al.[11], Miller et al.[14] and Ochiai et al.[26] presented observed SARS-CoV-2 infection prevalence ranging between 3 and 7%, with up to 6% of asymptomatic confirmed cases among screened population, which are similar to our findings.”
